# Rapid Visual Detection of Plasmodium Using Recombinase-Aided Amplification With Lateral Flow Dipstick Assay

**DOI:** 10.3389/fcimb.2022.922146

**Published:** 2022-06-24

**Authors:** Hong Lin, Song Zhao, Yanhong Liu, Lei Shao, Yuying Ye, Nizhen Jiang, Kun Yang

**Affiliations:** ^1^Jiangsu Province Blood Center, Nanjing, China; ^2^Key Laboratory of National Health and Family Planning Commission on Parasitic Disease Control and Prevention, Jiangsu Provincial Key Laboratory on Parasite and Vector Control Technology, Jiangsu Institute of Parasitic Diseases, Wuxi, China; ^3^Jiangsu Qitian Gene Technology Co., Ltd., Wuxi, China

**Keywords:** malaria, nucleic acid detection, plasmodium, recombinase-aided amplification, lateral flow dipstick

## Abstract

**Background:**

Malaria is a global public health problem. China has had no case of indigenous malaria since 2016. However, imported cases of malaria remain an issue among travelers, overseas workers, and foreign traders. Although these cases are always asymptomatic, if they donate blood, there is a great risk of transfusion transmitted-malaria (TTM). Therefore, blood banks need a rapid screening tool to detect *Plasmodium* species.

**Methods:**

We designed an assay using recombinase-aided amplification (RAA) and a lateral-flow dipstick (LFD) (RAA-LFD) to detect the 18S ribosomal RNA gene of *Plasmodium* species. Sensitivity was evaluated using a recombinant plasmid and Plasmodium genomic DNA. Specificity was evaluated using DNA extracted from the blood of patients with malaria or other infectious parasites. For clinical assessment, blood samples from patients with malaria and blood donors were evaluated.

**Results:**

The RAA-LFD assay was performed in an incubator block at 37°C for 15 min, and the amplicons were visible to the naked eye on the flow dipsticks within 3 min. The sensitivity was 1 copy/μL of recombinant plasmid. For genomic DNA from whole blood of malaria patients infected with *P. falciparum*, *P. vivax*, *P. ovale*, and *P. malariae*, the sensitivity was 0.1 pg/μL, 10 pg/μL, 10-100 pg/μL, and 100pg/μL, respectively. The sensitivity of this assay was 100pg/μL. No cross-reaction with other transfusion-transmissible parasites was detected.

**Conclusions:**

The results demonstrated that this RAA-LFD assay was suitable for reliable field detection of *Plasmodium* species in low-resource settings with limited laboratory capabilities.

## Introduction

Malaria is a life-threatening disease caused by infection with parasites of the *Plasmodium* family that is transmitted to humans through the bites of infected female *Anopheles* mosquitoes. Although core interventions have expanded greatly, malaria remains an unacceptably high threat as the gains are fragile and unevenly distributed. In 2020, the global malaria epidemic was exacerbated by the forced suspension of public health services in many malaria-endemic areas due to the COVID-19 pandemic. In 2020, there were an estimated 241 million cases of malaria and an increase in malaria-related deaths (627 000 deaths) (World Health Organization, 2021). The disease is endemic in all six WHO regions, although the burden is heaviest in the African Region, where an estimated 90% of malaria deaths occur (World Health Organization, 2021). Malaria outbreaks have occurred in countries that had previously been malaria free and resurgences in countries that had made progress in reducing malaria-related morbidity and mortality rates, highlighting the continual threat of the re-establishment and resurgence of malaria (Walker, 2020). Vigilance is required to ensure that areas of transmission are promptly identified and rapidly contained (Nasir et al., 2020). In non-endemic settings, malaria is mostly associated with travel or emigration from endemic areas. Some infected migrants or travelers returning from malaria-endemic areas are low parasite-density carriers who are asymptomatic and may become blood donors. It will cause less commonly transfusion transmitted-malaria (TTM) (Verra et al., 2018).

Blood donation centers throughout the world take various measures to prevent TTM, including health questionnaires, risk-based deferral, testing, and/or pathogen inactivation. Because parasitic infections are regional, most countries outside of these regions only use questionnaires. However, screening and detection are important for malaria elimination (WHO, 2015). In settings where malaria transmission is very low, active detection and investigation of infections in addition to free malaria care and notification at health facilities are important for eliminating residual transmission foci (WHO, 2012).

Polymerase chain reaction (PCR) can provide relatively high sensitivity for diagnostic testing, and some PCR-based tests have been developed to screen blood donors (Taylor et al., 2010; Lima et al., 2016). However, it is almost always cost prohibitive for routine screening (Taylor et al., 2010). Even in some developed countries, it is only selectively applied to screen Plasmodium risk blood donors (Shehata et al., 2004), not mention to being used in the underdeveloped malaria endemic countries. Nonetheless, TTM does occasionally happen in non-endemic areas (O'Brien et al., 2015). Therefore, accurate, sensitive, and affordable diagnostic methods suitable for use in remote areas are needed in the fight against malaria. In recent years, many isothermal amplification methods have been developed to detect Plasmodium infection, such as rolling circle amplification (RCA), loop mediated isothermal amplification (LAMP) (Loopamp™ Pf/Pv kit) (Selvarajah et al., 2020), recombinase polymerase amplification (RPA) (Kersting et al., 2014), and thermophilic helicase-dependent amplification (hHTA) (Li et al., 2013).

Recombinase-aided amplification (RAA) is a new isothermal amplification technology that is similar to RPA. This reaction system combines the recombinase UvsX and recombinase mediator protein UvsY from *Escherichia coli*, a single stranded DNA-binding (SSB) protein, and a DNA polymerase. UvsX and UvsY anneal primers to the template DNA, forming a protein-DNA complex that can bind to homologous sequences in a double-stranded DNA target. The untwisted double strands are bound by SSB to prevent DNA strand renaturation. Once the primer locates on the homologous sequence, a chain exchange reaction occurs that initiates DNA synthesis and chain extension by the DNA polymerase, exponentially amplifying the target region on the template (Lü et al., 2010). The amplification is performed at 37–42°C for 15–30 min and is a sensitive, rapid, low-cost isothermal amplification technology that does not require a thermal cycler. The amplicons can be detected by agarose gel electrophoresis (Feng et al., 2022), real-time fluorescence (Mu et al., 2021), or a lateral flow dipstick (LFD) (Zheng et al., 2021; Wang et al., 2022), which makes it possible to carry out simple, rapid, visual pathogen detection.

RAA combined LFD (RAA-LFD) assay has been developed as a rapid and visible method to detect several pathogens (Zhao et al., 2021; Li et al., 2022 ), but no study has yet reported the detection of parasites. In this study, we aimed to develop the assay for visual detection of *Plasmodium*. We evaluated the specificity and sensitivity of the assay and used the assay to test clinical samples.

## Materials and Methods

### Samples and DNA Extraction

Peripheral venous blood was collected into 5 mL test tubes containing ethylenediamine tetraacetic acid (EDTA). Malaria patients had been diagnosed in the outpatient clinic Jiangsu Institute of Parasite Diseases. Donors had been screened negative by rapid diagnosis test (RDT) before donation. Their blood samples had been detected by microscope and in-house RT-PCR. The in-house real-time PCR was performed as the same as a reference (Wang et al., 2014) in an ABI Prism 7500 Fast Real-time PCR system. All patients and donors signed consent information. After centrifugation and removal of plasma and buffy coat, DNA was extracted from 200 μL of packed red blood cells using the QIAamp® DNA Blood Mini Kit (Qiagen, Hilden, Germany) according to the manufacturer’s instructions.

### Generation of a Plasmid Standard

A recombinant plasmid containing a 450 bp fragment of the *P. falciparum* 18S ribosomal RNA gene (nt 181-1920, GenBank accession No. M19172.1) was constructed by cloning the PCR-amplified fragment of the 18S ribosomal RNA gene into the T-vector using the pGM-Simple-T-Kit (Zhongding Biotech [Jiangsu] Co., China). The plasmid DNA was quantified with a NanoDrop 2000 spectrophotometer (Thermo Fisher Scientific, Dreieich, Germany). The DNA copy number was calculated using the following formula: DNA copy number/μL = [6.02 × 10^23^ × plasmid concentration (ng/μL) × 10^-9^] / [DNA length (nt) × 660]. The constructed plasmids were verified by sequencing and stored at –20°C until use.

### Primer and Probe Design

The 18S ribosomal RNA gene was chosen as the target region using *Plasmodium* spp. sequences in the NCBI database (https://www.ncbi.nlm.nih.gov/nuccore). Several sets of RAA primers were designed within the conserved regions according to the principles of RAA primer and probe design (**Supplementary Table 1**). The primers and probe were labeled with fluorescent dyes for visualization on LFDs. The 5′-ends of the reverse primers were labeled with biotin, and the probe includes a 5′ antigenic label (6-carboxyfluorescein [FAM]), an internal abasic nucleotide analogue (dSpacer), and a polymerase extension-blocking group (SpC3) at the 3′ end. The primers and probe were synthesized by Genescript (Nanjing, China).

### RAA-LFD Assay Protocol

**Figure 1:**
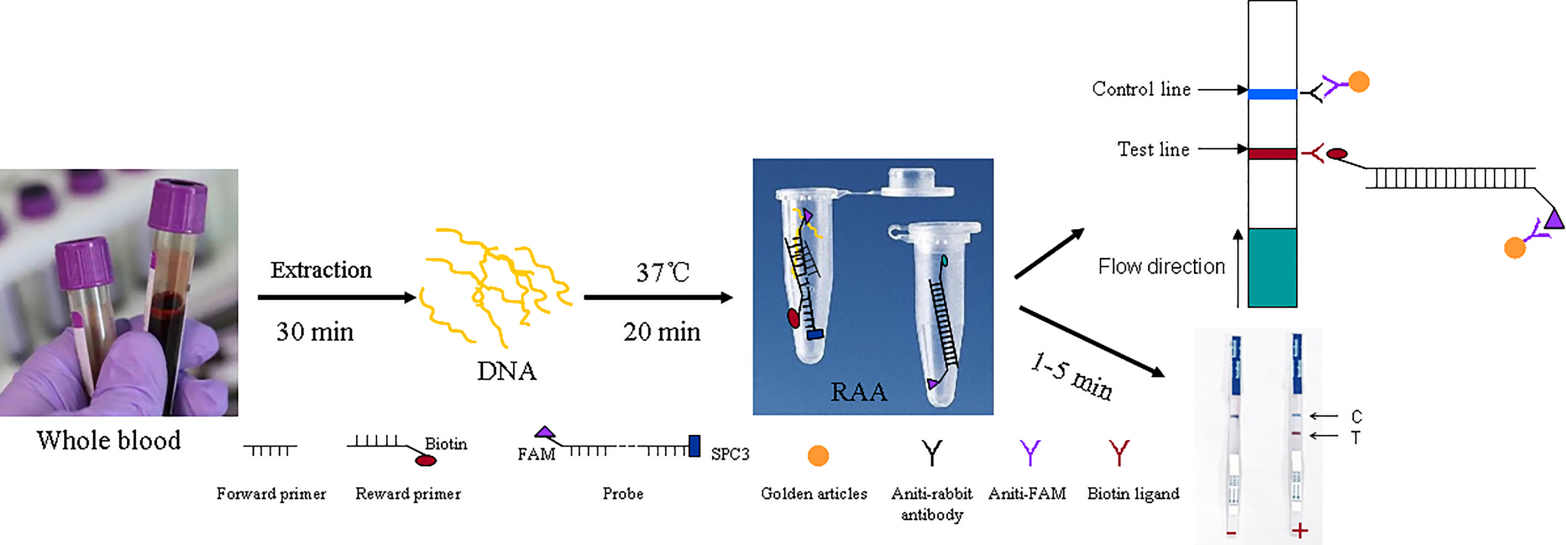
Flow chat of RAA-LFD. All work finish within 1 hour.

The RAA assay was performed using a commercial RAA kit (Cat. No. T00R01; Qitian Bio-Tech Co., Ltd., Jiangsu, China). Each RAA reaction contained 25 μl of reaction buffer, 10 mM ATP, 60 ng/µL recombinase, 50 ng/µL helper protein, 800 ng/µL SSB, 50 ng/µL DNA polymerase, 40 ng/μL endonuclease, 50 mM Tris (pH 7.4), 50 mM magnesium acetate, 5 mM dithiothreitol, 10% (w/v) PEG (15000–20000), 2.7 ng/μL creatine kinase, 10 μM FAM-labeled probe, 10 μM forward primer, 10 μM biotin-labeled reverse primer, 1 μL of template, and ddH_2_O to a total of volume 50 μL. After adding the ingredients, the reaction tube was shaken 10 times to mix. A positive control (the recombinant plasmid) and a negative control (H_2_O) were included in each run. The reactions were incubated in a 37°C water bath for 15–20 min. After amplification, 10 μL of each reaction was immediately removed and diluted with 90 μL of PBST in an Eppendorf tube and tested using a commercial LFD (Tiosbio, Beijing, China). The LFD was dipped into the tube, and the result was recorded when the positive control line was visualized (1–5 min). A positive result was recorded when both the test line (red) and control line (blue) appeared; while a negative result, only the control line appeared. When only the test line appeared, the result was considered invalid and was retested (**Figure 1**).

### Optimization of RAA-LFD Reaction Conditions

To determine the optimal reaction time, the reaction was stopped at 5, 10, 15, 20, 25, and 30 min after the addition of magnesium acetate by immediate dilution and analysis on the LFDs. The optimal temperature for the assay was determined by varying the temperature (30-40°C) of the RAA assay.

### Analytical Sensitivity and Specificity of the RAA-LFD Assay

To determine the sensitivity of the RAA-LFD assay, we evaluated this assay by three sources of DNA extracted from 1) recombinant plasmid and diluted from 10^3^ to 1^0^ copies/μL); 2) 200 μL of whole blood from patients who infected with *P. falciparum*, *P. vivax*, *P. malariae*, and *P. ovale* respectively and diluted from 10^3^ to 10^-2^ pg/μL; 3) 200μL whole blood mixed with cultured *P. falciparum* which were serially diluted (10^4^, 10^3^, 10^2^, 10, 1, 0.5, 0.25, parasites/μL). To evaluate the specificity of the assay, genomic DNA from patients infected with four *Plasmodium* species and other transfusion-transmissible parasites, including *Babesia microti*, *two Leishmania* species, *Anaplasma phagocytophilum*, and *Toxoplasma gondii*., were used as templates to be detected by RAA-LFD. These genomic DNA are saved in the Key Laboratory of National Health and Family Planning Commission on Parasitic Disease Control and Prevention in Jiangsu Institute of Parasitic Diseases. All RAA products were directly analyzed by LFD. All experiments were carried out in duplicate.

### Evaluation of the RAA-LFD Assay Using Clinical Samples

DNA was extracted from whole blood samples of 12 malaria patients and 100 blood donors and detected by RAA-LFD. The malaria patients were from the outpatient clinic department of Jiangsu Institute of Parasite Disease. The blood donors were malaria-risk and were international students from Africa and East Asia.

## Results

### Selection of Optimized Primers and Probe

To determine the optimal primer combination, RAA amplification was conducted as described in section 2.5 using various primer pairs. Nine primer pairs were tested, and all showed positive results with 1.0 × 10^2^ copies/μL plasmid. Only one primer pair (forward: 5′-CTTAATTTGACTCAACACGGGGAAACTCACT-3′ and reverse, 5′-biotin-TTATCGGAATTAACCAGACAAATCATATTCACG-3′) and probe: 5’-FAM-TTGACAGATTRAKAGCTCTTTCTTGATTTCTTG/idSp/ATGGTGATGCATGGC-C3spacer-3’, could amplify 10 copies/μL plasmid DNA, which was selected for subsequent experiments.

### Optimization of RAA-LFD Reaction Conditions

**Figure 2:**
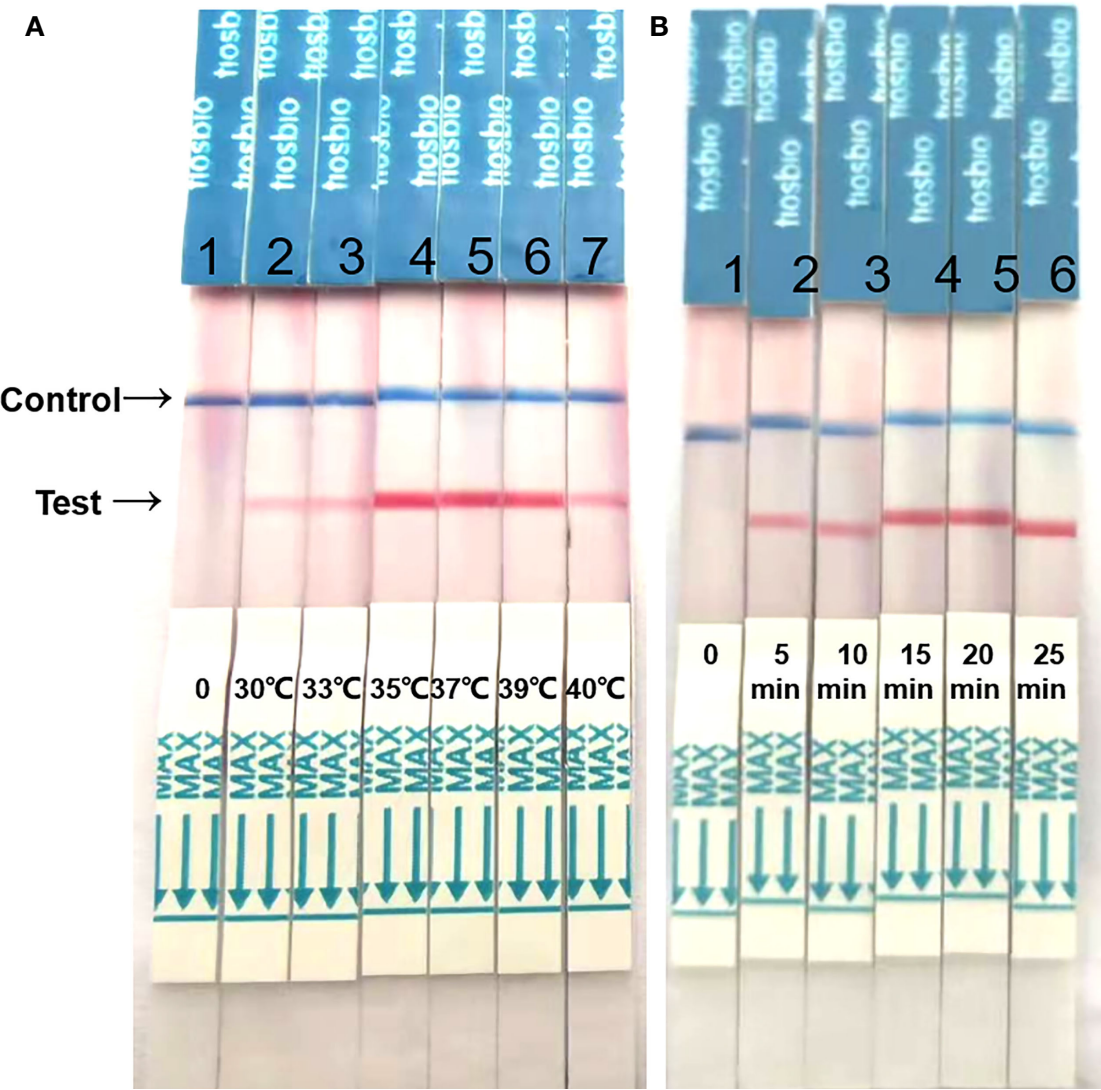
Optimization of reaction temperature and time. **(A)** The RAA-LFD works effectively in a broad range of constant reaction temperatures. **(B)** After 20 min of isothermal amplification reaction, the test line is visible on the test strip. Including the incubation of 5 min the whole assay time of the RAA-LFD is less than 20 min.

The reaction time and temperature were optimized. Compared with the results, at 37°C and 39°C, the band intensity was similar, however at 37°C, the band became visible after 5 min, and after 15 min, the band was clear and the density was darker than that at 39°C. So, the optimum reaction conditions were a reaction temperature of 37°C and a 15 min reaction time (**Figure 2**).

### Analytical Sensitivity of the RAA-LFD Assay

**Figure 3:**
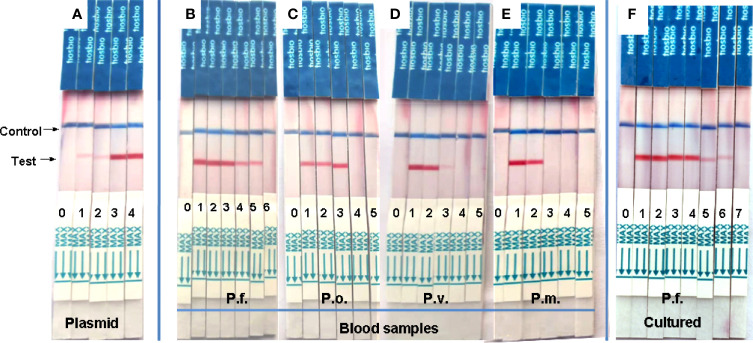
Analytical sensitivity of RAA-LFD assay. **(A)** was a serial dilution the recombinant plasmid from10^3^ to 10^0^ copies/μL. The sensitivity was 1 copies/μL. 0-H_2_O, 1-10^0^ copies/μL, 2-10^1^ copies/μL, 3-10^2^ copies/μL, 4-10^3^ copies/μL. **(B–E)** were gradient dilution of genomic DNA from *P. faciparlum* (P.f., 0.1pg/μL), *P. oval* (P.o., 10 pg/μL), *P. vivax* (P.v., 10-100 pg/μL) and *P. malariae* (P.m., 100pg/μL) DNA from 200μL whole blood, respectively. 0-H_2_O, 1-10^3^ pg/μL, 2-10^2^ pg/μL, 3-10 pg/μL, 4-1pg/μL, 5-10^-1^ pg/μL, 6-10^-2^ pg/μL. **(F)** was the parasite density of cultured P. faciparlum (P.f.) and the sensitivity was 0.5 parasites/μL. 0-H_2_O, 1-10^4^ parasites/μL, 2-10^3^ parasites/μL, 3-10^2^ parasites/μL, 4-10 parasites/μL, 5-1 parasite/μL, 6-0.5 parasite/μL, 7-0.25 parasite/μL.

After optimizing the reaction conditions, we used various concentrations (1000, 100, 10, 1, and 0.1 copies/μL) of a recombinant plasmid as a template to assess sensitivity, and the results showed that the sensitivity of this system was 1 copy/μL (**Figure 3A**). The values for genomic DNA of *P. falciparum*, *P. oval*, *P. vivax*, and *P. malariae* from whole blood were 0.1 pg/μL (**Figure 3B**), 10 pg/μL (**Figure 3C**), 10–100 pg/μL (**Figure 3D**), and 100 pg/μL (**Figure 3E**), respectively. The detection sensitivity of cultured *P. falciparum* was 0.5 parasites/μL in 200μL whole blood (= 2.5 parasites/mL) (**Figure 3F**).

### Analytical Specificity of the RAA-LFD Assay

**Figure 4:**
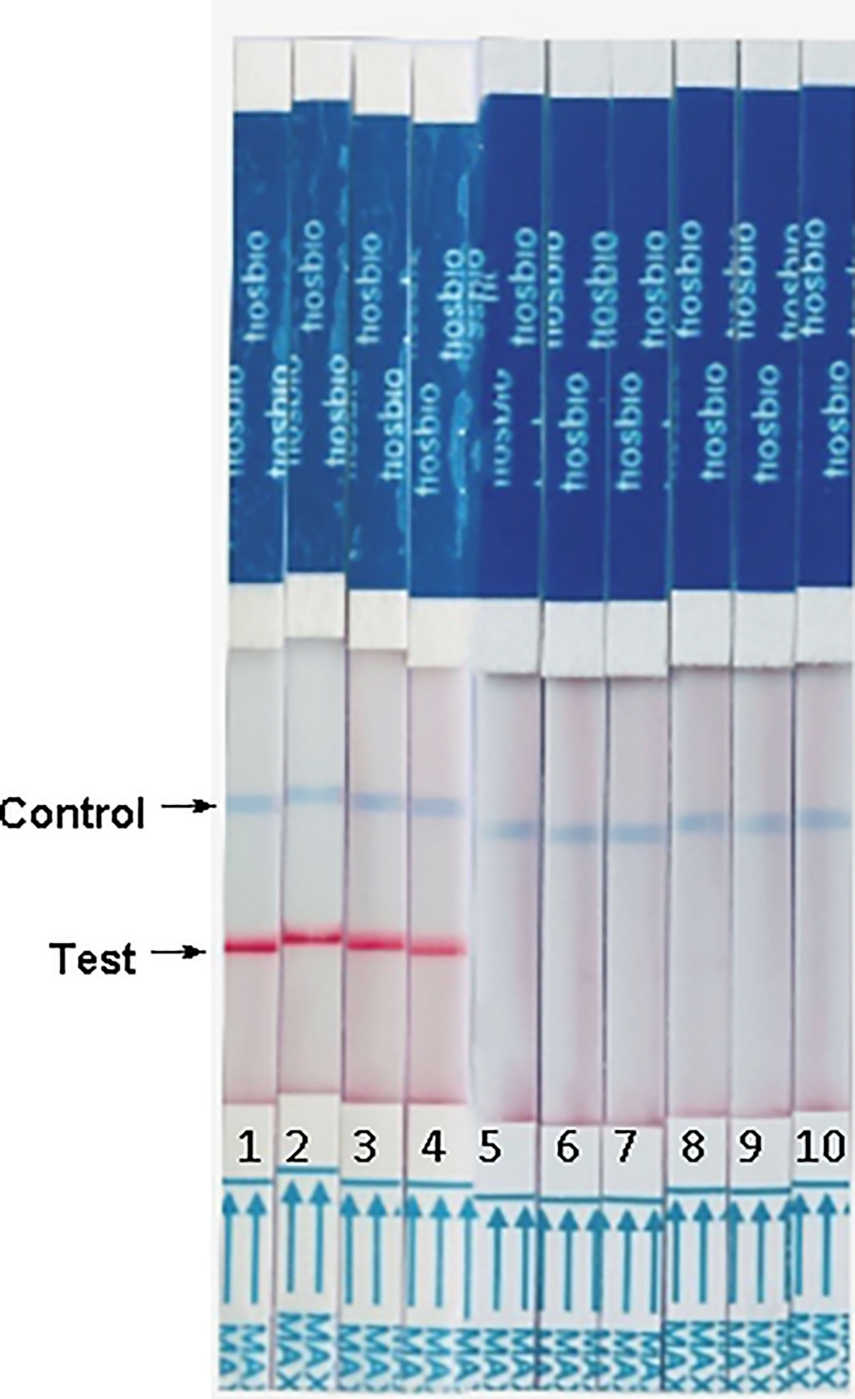
Analytical specificity of reverse transcription RAA-LFD assay. From left to right, the test samples are 1-*P. vivax*, 2-*P. malariae*, 3-*P. falciparum*, 4-*P. ovale*, 5-*Leishiamnia donovani*, 6-*Leishiamnia infatum*, 7-*Toxoplasm gondii*, 8-*Babesia microti*, 9-*Anaplasma phagocytophilum* and 10-negative control, H_2_O.

We conducted specificity experiments using transfusion-transmissible parasites that can inhabit the cellular components of blood, including *Plasmodium* species, *Babesia* species, *Leishmania* species, *Anaplasma phagocytophilum*, and *Toxoplasma gondii*. Bands only appeared in reactions with *Plasmodium* species (1–4 in **Figure 4**), and no bands in reactions with the other parasites (5–9 in **Figure 4**). These results indicate that the RAA-LFD assay can effectively distinguish *Plasmodium* species from other parasites and does not cross-react with them, thus the assay exhibited high specificity for the detection of *Plasmodium* species.

### Evaluation of the RAA-LFD Assay Using Clinical Samples

**Figure 5:**
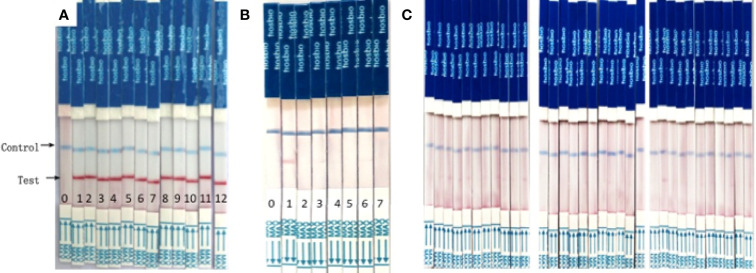
RAA/LFD results for a selection of clinical samples. **(A)** malaria patients. 0- H_2_O, 1-12 DNA extracted from malaria patients. **(B, C)**. foreign university student blood donors. In **(B)**, 0-negative control, H_2_O, 1- asymptomatic PCR positive donors, 2-6, other PCR negative blood donors. In **(C)**. The results of RAA-LFD were all negative.

We evaluated the RAA-LFD assay using previously defined clinical samples from 12 malaria patients and malaria-risk blood donors confirmed by in-house PCR. **Figure 5A** showed that all malaria patients could be diagnosed. **Figures 5B, C** were the screening results of blood donors. A malaria asymptomatic donor could be screened in **Figure 5B**. **Figure 5C** illustrated some of RT-PCR-negative samples were detected by RAA-LFD and no positive results was found.

## Discussion

In malaria-endemic areas, natural infection occurs *via* mosquito bite rather than from blood transfusion. Many blood donors and transfusion patients are already infected with or are at high risk of infection with malaria, even if they are asymptomatic. In these areas, Giemsa-stained thick films or rapid diagnostic tests (RDTs) for malarial antigens are used to identify those with higher levels of parasitemia. However, in non-endemic countries, appropriate donor deferral guidelines are used to minimize the occurrence of TTM. However, these guidelines are insufficient to prevent TTM (Holtzclaw et al., 2016), because migrants and returning leisure and business travelers often show very low parasitemia and could not be timely or properly deferred. Therefore, highly sensitive, cost-effective laboratory screening for high-risk donors is needed.

Molecular detection methods for malaria research, screening high-risk populations, and diagnosis in clinical care have improved remarkably over the past two decades, and these methods usually have higher sensitivities and specificities than microscopy and RDT (Naing et al., 2022). The products of nucleic acid amplification can be detected using techniques such as gel electrophoresis, turbidity measurement of magnesium pyrophosphate, and fluorescent dye methods among others. Since some methods, are tedious or require an expensive machine, such as electrophoresis and fluorescent dyes. Faster and simpler methods for detection have been developed, such as chromatographic LFDs. Here, we developed a highly sensitive, specific, molecular diagnostic method that can detect four species of *Plasmodium* for the screening of blood donors from East Asia and Africa. Specifically, we used biotin-labeled reverse primers and a FAM-labeled probe to generate biotin- and FAM-labeled RAA products. We then visualized the product as it flowed through the anti-FAM antibody region of an LFD (**Figure 1**). This method allows direct visual observation of the results without any expensive dedicated equipment and is suitable for on-site screening in remote areas with malaria outbreaks.

We evaluated the sensitivity of the assay using 10-fold serial dilutions of a plasmid, which indicated that this RAA-LFD method can detect 1 copy/μL in 20 min. Depending on the strain, the copy number of the 18S ribosomal RNA gene per parasite varies from five to eight in *P. falciparum* and from four to eight in *P. vivax* and *P. ovale* (Mercereau-Puijalon et al., 2002). Theoretically, 125–200 parasites/mL could be detected by our RAA-LFD assay. The sensitivity was similar to that of a novel FRET real-time PCR assay (199.97 parasites/mL) (Schneider et al., 2021). For gene DNA extracted from patients, the detection limit of a RPA-LFD assay was reported to be as low as 100 fg of genomic *P. falciparum* DNA, corresponding to approximately four parasites per reaction or 20 parasites/mL (Kersting et al., 2014). Our RAA-LFD assay could also detect the same low concentration of gene DNA (**Figure 3B**), which was lower than that of another RPA-LFD assay (64 parasites/mL) (Lalremruata et al., 2020). Since only *P. falciparum* could be cultured, the senstitivity of *P. falciparum* parasite also was determined and it was 0.5 parasites/μL (=500 parasites/mL) (**Figure 3F**). We also evaluated the RAA-LDF assay by detect a known copy number *P. falciparum* and the sensitivity was 1 copy/μL of *P. falciparum* DNA (**Supplementary Figure 2**), which is the same as the sensitivity of a LAMP kit (Azam et al., 2021). Furthermore, The RAA-LFD assay could detect *P. falciparum* DNA in an asymptomatic blood donor sample that was negative by microscopy (which can detect as few as 50 *P*. *falciparum* parasites/mL blood) (**Figure 5B**). Hence, the sensitivity of the RAA-LFD assay is considerably high.

Unlike most molecular assays that target one species of *Plasmodium*, our RAA-LFD assay was developed to screen for plasmodium infection among blood donors, thus the primers were designed to target four common *Plasmodium* species. Our analysis showed that the assay could detect genomic DNA of four *Plasmodium* species, although the sensitivity varied—the sensitivity was the highest for *P. falciparum* and the lowest for *P. malariae*. This result agreed with that of RT-PCR (Wang et al., 2014) and FRET real-time PCR (Schneider et al., 2021) assays. However, in an assay combining qRT-PCR and high-resolution melting, the LOD for *P. vivax* was 1 copy, whereas it was 10 copies for *P. ovale* and 100 copies for both *P. falciparum* and *P. malariae* (Chua et al., 2015). The 18S rRNA gene is stable and conserved in all *Plasmodium* species; thus, the reason for the difference in sensitivity needs to be further studied.

There are four parasitic diseases that can be transmitted *via* transfusion: babesiosis, Chagas disease, leishmaniasis, and malaria, which are caused by *Babesia* spp., *Trypanosoma cruzi*, *Leishmania* spp., and *Plasmodium* spp., respectively (Leiby et al., 2019). In some countries, these parasites may be a risk to blood safety. These diseases have similar clinical manifestations, and in some cases the parasites are biologically similar to those that cause malaria parasites. In particular, *Babesia* spp. cannot be differentiated from the ring form of *P. falciparum* under a microscope (Zhou et al., 2014), so molecular methods may be more effective for identification. However, for some methods targeting the 18S rRNA gene, positive results were observed not only for *Plasmodium* but also several other homologous eukaryotes (Rougemont et al., 2004). We evaluated the specificity of our RAA-LFD assay using the above mentioned parasites and observed no cross-reaction, demonstrating that the assay has good specificity that can avoid misdiagnosis.

Twelve random clinical samples from malaria patients who were diagnosed by the Jiangsu Institute of Parasite Diseases were used in this study. The results of the RAA-LFD assay were all positive (**Figure 5A**). Jiangsu province was certified as malaria-free by WHO in 2021 (Cao et al., 2022), despite some cases of TTM which all caused by imported plasmodium in prior years (Zhang et al., 2020). In one of TTM cases, the blood donor was RDT negative and microscope negative, but RT-PCR positive with a high Ct value was always asymptotic even at the one-year follow-up (Case 7 in Lin, 2021). Since then, all the blood samples from malaria-risk blood donors have been sent to Jiangsu Institute of Parasite Disease to avoid TTM. Analysis of genomic DNA from this blood donor using our RAA-LFD assay yielded a positive result (**Figure 5B**); **Figure 5C** illustrated negative results by RAA-LFD which agreed with the results of in-house PCR, which demonstrated that this method could be used for screening blood donors.

In conclusion, we developed a novel *Plasmodium* spp. detection assay, RAA-LFD, which enables rapid and easy visualization of test results in 30 min. The assay exhibited excellent sensitivity and specificity for a variety of samples, including submicroscopic samples. Our results indicate that the developed RAA-LFD assay is a reliable and portable sceening tool for detecting *Plasmodium* spp. and is therefore suitable for blood screening even in under-equipped laboratories and primary care facilities.

## Data Availability Statement

The original contributions presented in the study are included in the article/**Supplementary Material**. Further inquiries can be directed to the corresponding authors.

## Ethics Statement

The studies involving human participants were reviewed and approved by Jiangsu Province Blood center/Jiangsu Institute of Parasite Disease. The patients/participants provided their written informed consent to participate in this study.

## Author Contributions

HL, SZ, and KY contributed to the research design. HL, SZ, and YL developed and evaluated the RAA-LFD assay. SZ and KY provided clinical patient samples. YL, LS, YY and NJ performed the experiments. HL wrote the manuscript, SZ, NJ, and KY supervised the study and edited the manuscript. All authors contributed to the article and approved the submitted version.

## Funding

This work was supported by Social Development Project (No.BE2019755) and International Technology Corporation Project (No.BZ2020003) from the Department of Science and Technology of Jiangsu Province.

## Conflict of Interest

YL is employed by Jiangsu Qitian Gene Technology Co., Ltd.

The remaining authors declare that the research was conducted in the absence of any commercial or financial relationships that could be construed as a potential conflict of interest.

## Publisher’s Note

All claims expressed in this article are solely those of the authors and do not necessarily represent those of their affiliated organizations, or those of the publisher, the editors and the reviewers. Any product that may be evaluated in this article, or claim that may be made by its manufacturer, is not guaranteed or endorsed by the publisher.
