# Supplementary material for: Rapid Visual Detection of Plasmodium Using Recombinase-Aided Amplification With Lateral Flow Dipstick Assay
Source: Front Cell Infect Microbiol. 2022 Jun 24;12:922146. doi: 10.3389/fcimb.2022.922146 (PMC9263184; doi:10.3389/fcimb.2022.922146)
Supplement: Supplementary file 1 [file DataSheet_1.doc]

Supplementary Figure


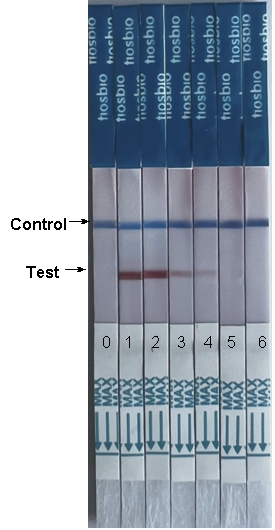


Figure 1 Limitation of detection (LOD) of copy numbers of *Plasmodium faciparlium* DNA by RAA-LFD. 0-H_2_O, 1-10^3^ copies/μL, 2-10^2^ copies/μL, 3-10^1^ copies/μL, 4- 1copy/μL, 5-10^-1^ copies/μL, 6-10-^2^ copies/μL. The LOD was 1copy/μL.
