# Supplementary material for: Rapid Visual Detection of Plasmodium Using Recombinase-Aided Amplification With Lateral Flow Dipstick Assay
Source: Front Cell Infect Microbiol. 2022 Jun 24;12:922146. doi: 10.3389/fcimb.2022.922146 (PMC9263184; doi:10.3389/fcimb.2022.922146)
Supplement: Supplementary file 2 [file Table_1.doc]

**Supplement TABLE 1** Sequences of primers and probe in this study

| **Primers and probe** | | **Sequence (5’-3’)** |
| --- | --- | --- |
| Primers | F1 | **CTTAATTTGACTCAACACGGGGAAACTCACT** |
|  | R1 | Biotin-CTAATTAGCAGGTTAAGATCTCGTTCGTTATCG |
|  | F2 | CTTCCTTCAGTRCCTTATGAGAAATCAAAGTC |
|  | R2 | Biotin-CCGCTAATTAGCAGGTTAAGATCTCGTTCG |
|  | F3 | CGTGGAGCTTGCGGCTTAATTTGACTCAACA |
|  | R3 | **Biotin-TTATCGGAATTAACCAGACAAATCATATTCACG** |
| Probe | | Labeled before:  TTGACAGATTRAKAGCTCTTTCTTGATTTCTTGGATGGTGATGCATGGC  Labeled after:  FAM-TTGACAGATTRAKAGCTCTTTCTTGATTTCTTG/idSp/ATGGTGATGCATGGC-C3spacer |
